# Role of the cerebro-placental-uterine ratio in predicting adverse perinatal outcome in low-risk pregnancies at term

**DOI:** 10.1007/s00404-022-06733-8

**Published:** 2022-08-30

**Authors:** Oliver Graupner, Markus Meister, Linda Lecker, Sepideh Karim-Payab, Cordula Franz, Juliane Carow, Christian Enzensberger

**Affiliations:** 1 Department of Obstetrics and Gynecology, University Hospital Aachen, RWTH Aachen University, Pauwelsstraße, 30 52074 Aachen, Germany; 2 Department of Obstetrics and Gynecology, University Hospital Rechts Der Isar, Technical University, Munich, Germany

**Keywords:** Full term, Late term, Cerebroplacental ratio, Cerebroplacental-uterine ratio, Uterine artery doppler, Adverse perinatal outcome

## Abstract

**Purpose:**

The cerebroplacental ratio (CPR) is associated with adverse perinatal outcome (APO) in low-risk pregnancies near term. A Doppler parameter, which also includes information from the uterine vessels could potentially improve detection of subclinical placental dysfunction. The aim of this study is to investigate the performance of cerebro-placental-uterine ratio (CPUR) related to APO prediction in low-risk term pregnancies in > 40 + 0 weeks.

**Methods:**

This is a retrospective cohort study. All low-risk pregnancies in which feto-maternal Doppler was examined from 40 + 0 weeks and an appropriate for gestational age fetus was present were included. ROC (receiver operating characteristic curves) analyses were performed to assess the predictive value of CPUR. The presence of at least one of the following outcome parameters was defined as composite APO (CAPO): operative delivery (OD) due to intrapartum fetal compromise (IFC), admission to the neonatal intensive care unit, umbilical cord arterial pH ≤ 7.15, 5 min APGAR ≤ 7.

**Results:**

A total of *n* = 114 cases were included. Mean gestational age at examination and delivery were 40 + 3 weeks and 40 + 6 weeks, respectively. Overall, CAPO occurred in 38 of 114 cases (33.3%). ROC analyses showed a significant association of CPUR (AUC = 0.67, *p* = 0.004) and CPR (AUC = 0.68, *p* = 0.002) with CAPO. Additionally, CPUR (AUC = 0.64, *p* = 0.040) showed a predictive value for OD due to IFC.

**Conclusion:**

The CPUR in > 40 + 0 weeks showed a predictive value for CAPO and OD due to IFC in low-risk pregnancies. However, the extent to which CPUR can be used to optimize delivery management warrants further investigations in prospective interventional studies.

**Supplementary Information:**

The online version contains supplementary material available at 10.1007/s00404-022-06733-8.

## What does this study add to the clinical work

**Table:** 

The cerebro-placental-uterine ratio (CPUR) is a potential marker of placental dysfunction including fetal as well as maternal Doppler information in one ratio. The CPUR in >40+0 weeks showed a mild predictive value for adverse perinatal outcome and operative delivery due to intrapartum fetal compromise in low-risk pregnancies.		

## Introduction

Feto-maternal Doppler sonography has achieved a high status in diagnosis, therapy, and monitoring of high-risk pregnancies in prenatal medicine. Especially the cerebroplacental ratio (CPR), the ratio of pulsatility index (PI) of middle cerebral artery (MCA) and umbilical artery (UA) has become the focus of current studies in recent years as a marker of fetal well-being and an adverse perinatal outcome (APO) in appropriate for gestational age (AGA) fetuses [1–8]. However, the CPR includes only fetal Doppler information and shows a moderate predictive power regarding APO [9, 10].

Evaluation of uterine Doppler ultrasound in the third trimester in low-risk AGA fetuses demonstrated a significant association with uteroplacental dysfunction in form of an increased uterine vascular resistance (mean uterine artery PI: mUtA‐PI) and fetal cerebral blood flow redistribution (“brain-sparing”) reflected by low CPR values [1]. Similarly, recent published data on the role of uterine Doppler in low-risk term pregnancies in early labor showed an association between raised mUtA-PI with an higher incidence of obstetric intervention due to fetal distress [11]. Hence, a Doppler parameter combining all these information from the uterine, placental, and fetal vessels could potentially improve detection of subclinical placental dysfunction (PD) at term compared to CPR even more [12].

The cerebro-placental-uterine ratio (CPUR: CPR/mUtA-PI) is a poorly studied parameter, which includes fetal as well as maternal Doppler information. The idea of integrating the maternal Doppler ultrasound information into APO risk assessment gave the occasion to first studies, which investigate the role of CPUR in AGA and small for gestational age (SGA) fetuses [13–15]. This gain in information could lead to a better APO prediction in low-risk term pregnancies. In planning the optimal time of delivery for pregnancies beyond 40 + 0 weeks, in which PD plays a decisive role, and in achieving a better perinatal outcome, CPUR could be a useful tool.

The aim of this work is to investigate the performance of CPUR for APO prediction in low-risk pregnancies beyond 40 + 0 weeks of gestation.

## Methods

### Study population

This is a retrospective, single-center cohort study. Low-risk pregnancies in which feto-maternal Doppler was examined from 40 + 0 weeks and an AGA–fetus (estimated fetal weight ≥ 10th and < 90 percentile) was present were included. Data were obtained between September 2019 and December 2021. Cases with the evidence of chromosomal or morphological anomalies, twin pregnancies, or other conditions with possible effect on fetal hemodynamics such as pre-eclampsia or endocrine disorders (e.g. maternal diabetes) were excluded from analysis. All term pregnancies were monitored and treated (offer of induction of labor at 41 + 0 weeks of gestation, indication for induction of labor at 41 + 3 weeks of gestation at the latest) following the recommendations of national guidelines [16].

### Feto-maternal Doppler assessment

Feto-maternal Doppler examinations were performed using a Voluson E10 and E8 (GE Medical Systems, Solingen, NRW, Germany) with a 2–8 MHz convex probe including umbilical artery (UA) pulsatility index (PI), middle cerebral artery (MCA) PI and mean uterine artery (mUtA) PI in all cases. Doppler measurements were performed by trained operators of our institution of prenatal diagnostics and obstetrics following the recommendations of the International Society of Ultrasound in Obstetrics and Gynecology (ISUOG) and national guidelines [17, 18]. CPR was calculated as MCA-PI/UA-PI. mUtA-PI was calculated as the average PI of right and left uterine arteries (UtA). CPUR was calculated as CPR/mUtA-PI. Mean UtA-PI was defined as abnormal when it was > 95th centile [19]. CPR was defined as abnormal when it was < 5th centile [20]. In case of more than one Doppler examination, the closest examination to delivery was included.

We routinely determine the CPR in low-risk pregnancies during full term (39 + 0–41 + 0 weeks) in our department. In the case of a CPR abnormality (< 5th centile) between 40 + 0 and 41 + 0, we understand this as a risk factor for perinatal morbidity and mortality. Therefore, we discuss induction of labor with the patients in informed consent on the basis of the current evidence for CPR in the low-risk collective (and even on data from the ARRIVE study [21] independent of the CPR). From 41 + 0 we clearly recommend the induction of labor in the event of CPR abnormalities (also here based on the SWEPIS study [22] independent of the CPR).

### Statistical analysis

IBM SPSS statistics (Version 27.0 for Windows) was used for statistical analysis. Receiver operating characteristic curves (ROC) analyses were performed to assess the predictive value of CPUR, CPR and mUtA-PI with respect to the occurrence of adverse perinatal outcome (APO). The presence of at least one of the following APO parameters was defined as composite APO (CAPO):

- Emergency operative delivery (OD) due to intrapartum fetal compromise (IFC).
- Admission to the neonatal intensive care unit (NICU).
- Umbilical cord arterial pH ≤ 7.15.
- 5 min APGAR ≤ 7.

The diagnosis of IFC was made based on abnormal fetal heart rate (FHR) patterns and/or pH value ≤ 7.20 of fetal blood sampling (scalp). Abnormal FHR was defined as a pathological cardiotocography (CTG) according to the International Federation of Gynecology and Obstetrics (FIGO) criteria [23]. OD was defined as cesarean section (CS) or instrumental vaginal delivery (IVD). A threshold value for CPUR above which a CAPO was achieved was deliberately omitted, since too few cases were available for this purpose. To detect possible associations between APO criteria and the used Doppler parameters linear regression analysis using Pearson correlation were performed. Unstandardized regression coefficient b was reported. All accomplished statistical tests were performed with a significance level of 0.05. The data were validated by using double data entry. Descriptive statistics are presented as means ± 1 standard deviation.

## Results

This study included *n* = 114 low-risk pregnancies, which were examined after 40 + 0 weeks of gestation. Mean time of examination was 40 + 3 weeks of gestation. Mean time of delivery was 40 + 6 weeks of gestation. All included Doppler measurements were complete, allowing calculation of CPUR. Overall, CAPO occurred in 34 of 114 cases (33.3%). APO-frequencies and baseline characteristics of the study cohort are displayed in Table 1.

**Table 1:** APO–frequencies and cohort characteristic of *n* = 114 low-risk pregnancies

	Non-CAPO	CAPO	*p*-value
Maternal age [years] (mean)	30.91 ± 4.7	30.89 ± 4.6	0.959
BMI [kg/m^2^] (mean)	24.49 ± 4.4	25.77 ± 4.4	0.082
GA at examination [weeks] (Mean)	40 + 3 ± 3	40 + 3 ± 2	0.788
GA at delivery [weeks] (mean)	40 + 6 ± 3	40 + 6 ± 3	0.781
Birth weight [kg] (mean)	3597 ± 401	3554 ± 408	0.534
Primiparous	45/76 (59.2%)	26/38 (68.4%)	0.339
IVF/ICSI	2/76 (2.6%)	2/38 (5.3%)	0.472
Previous cesarean section	5/76 (6.6%)	6/38 (15.7%)	0.116
CPR < 5. Percentile	1/76 (1.3%)	5/38 (13.2%)	0.008
mUtA-PI > 95. Percentile	7/76 (9.2%)	6/38 (15.8%)	0.298
SDP < 2 cm	8/76 (10.5%)	2/38 (5.3%)	0.349
Induction of labour*	25/76 (32.9%)	15/38 (39.5%)	0.488
Dinoproston	5/76 (6.6%)	1/38 (2.6%)	0.374
Misoprostol	20/76 (26.3%)	12/38 (31.6%)	0.555
Minprostin	2/76 (2.6%)	2/38 (5.3%)	0.472
Mechanical	0/76 (0.0%)	0/38 (0.0%)	–
Spontaneous vaginal delivery	62/76 (81.6%)	15/38 (39.5%)	< 0.001
Secondary cesarean section	13/76 (17.1%)	14/38 (36.8%)	0.019
Emergency cesarean section**	0/76 (0.0%)	4/38 (10.5%)	< 0.001
Vaginal operative delivery	0/76 (0,0%)	9/38 (23.7%)	< 0.001
NICU	0/76 (0,0%)	12/38 (31.6%)	< 0.001
pH ≤ 7,15	0/76 (0.0%)	18/38 (47.4%)	< 0.001
5-Minute APGAR ≤ 7	0/76 (0.0%)	2/38 (5.3%)	0.044

*GA* gestational age, *CPR* cerebroplacental ratio, *mUtA-PI* mean uterine artery pulsatility index, *APO* adverse perinatal outcome, *CAPO* combined adverse perinatal outcome, *NICU* neonatal intensive care unit]

*n = 2 patients received two induction methods, so total number of birth induction doesn’t match the individual frequencies

**Number of emergency cesarean sections from *n* = X secondary cesarian sections

**Fig. 1:**
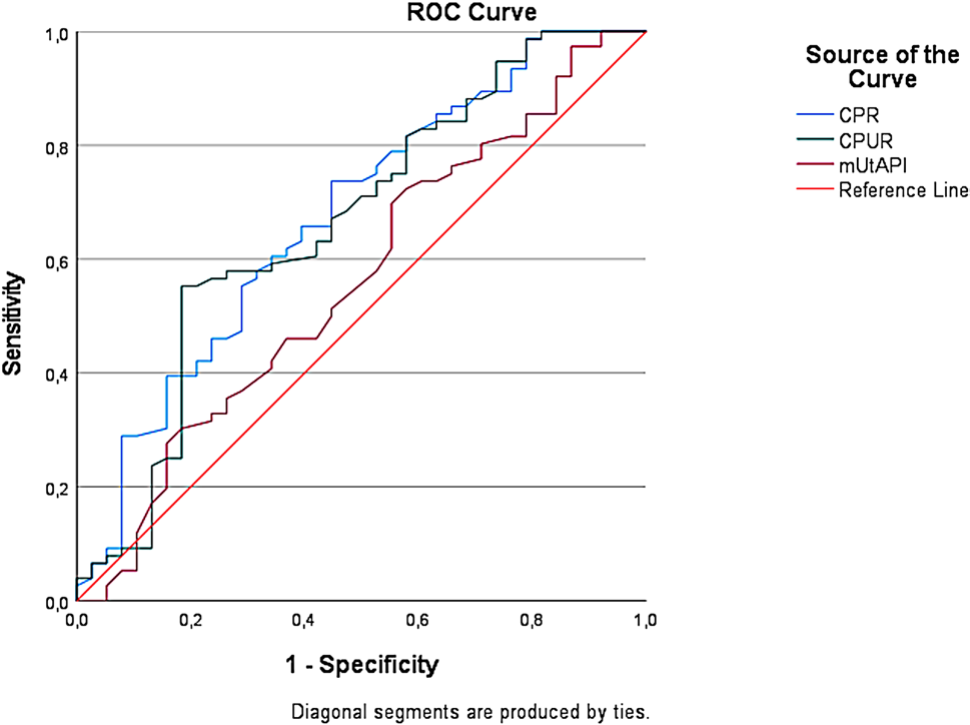
Graphical results of ROC—analysis. Predictive performance of CPR (blue line), CPUR (green line) and mUtA-PI (red line) for the occurrence of CAPO. *ROC* receiver operating characteristics, *CPR* cerebroplacental ratio, *CPUR* cerebroplacental-uterine ratio, *mUtA-PI* mean uterine artery pulsatility index, *CAPO* combined adverse perinatal outcome

**Fig. 2:**
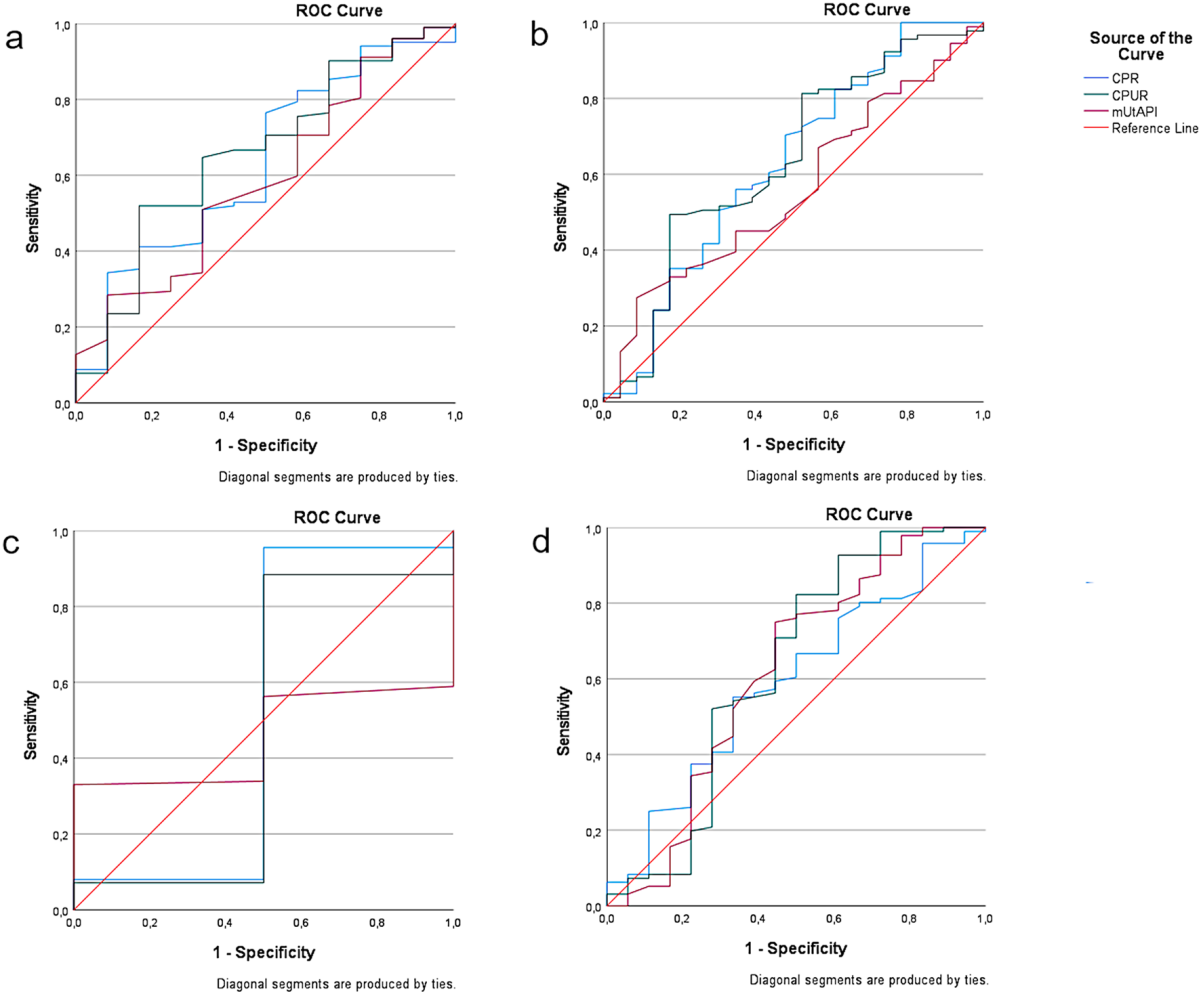
Graphical results of ROC—analysis. Predictive performance of CPR (blue line), CPUR (green line) and mUtA-PI (red line) for the occurrence of the individual APO´s (**a**–**d**). **a** APO NICU, **b** APO OC for IFC, **c** APO APGAR ≤ 7, **d** APO pH ≤ 7.15. *ROC* receiver operating characteristics, *CPR* cerebroplacental ratio, *CPUR* cerebroplacental-uterine ratio, *mUtA-PI* mean uterine artery pulsatility index, *CAPO* combined adverse perinatal outcome

ROC analyses showed a significant association of CPUR (AUC = 0.67, 95% CI 0.55–0.78, *p* = 0.004) and CPR (AUC = 0.68, 95% CI 0.57–0.78, *p* = 0.002) with CAPO. mUtA-PI (AUC = 0.56, 95% CI 0.45–0.68, *p* = 0.280) showed no significant association with CAPO. Considering the predictive value of CPUR, CPR and mUtA-PI for the individual APO indices, only in the case of CPUR (AUC = 0.64, 95% CI 0.50–0.77, *p* = 0.040) a significant predictive value for OD for IFC could be demonstrated. No association could be demonstrated for any other APO criteria. The results of ROC are displayed in Table 2 as well as Figs 1 and 2.

**Table 2:** Numerical results of ROC—analysis. Predictive performance of CPR, CPUR and mUtA-PI for the occurrence of CAPO and the individual APO-parameters

Outcome	Variables	AUC	Std. error	Asymp. Sig	Asymp. 95% confidence interval	
					Lower bound	Upper bound
CAPO	CPR	0.68	0.05	0.00	0.57	0.78
	CPUR	0.66	0.06	0.00	0.55	0.78
	mUtAPI	0.56	0.06	0.28	0.45	0.68
APO pH ≤ 7.15	CPR	0.59	0.07	0.23	0.46	0.73
	CPUR	0.63	0.09	0.09	0.45	0.80
	mUtAPI	0.62	0.09	0.12	0.45	0.78
APO OD for IFC	CPR	0.65	0.07	0.06	0.49	0.77
	CPUR	0.64	0.07	0.04	0.50	0.77
	mUtAPI	0.56	0.06	0.38	0.43	0.69
APO APGAR	CPR	0.52	0.31	0.93	0.00	1.00
	CPUR	0.48	0.29	0.91	0.00	1.00
	mUtAPI	0.46	0.10	0.83	0.27	0.64
APO NICU	CPR	0.63	0.08	0.13	0.47	0.80
	CPUR	0.66	0.08	0.07	0.50	0.82
	mUtAPI	0.59	0.09	0.31	0.43	0.76

*ROC* receiver operating characteristics, *AUC* area under the curve, *CPR* cerebroplacental ratio, *CPUR* cerebroplacental-uterine ratio, *mUtA-PI* mean uterine artery pulsatility index, *CAPO* combined adverse perinatal outcome, *APO* adverse perinatal outcome, *OD for IFC* operative delivery for intrapartum fetal compromise, *NICU* neonatal intensive care unit

Linear regression analysis revealed a significant association between CPUR, CPR as well as mUtA-PI and umbilical arterial pH. CPUR achieved the highest correlation coefficient here (*b* = 3.52; *p* = 0.005). Figure S1 shows the graphical representation of the linear regression analyses. The corresponding numerical representation can be found in Table S1.

## Discussion

The aim of the study was to investigate the performance of CPUR regarding APO prediction in low-risk pregnancies at term compared to the established feto-maternal Doppler indices CPR and mUtA-PI. We observed a significant association of low CPUR and CPR with CAPO, although the overall predictive power of CPR and CPUR was mild. No significant advantage of CPUR compared to CPR in the overall CAPO prediction rate could be found. A predictive value of CPUR regarding OD for IFC could be demonstrated. Linear relationship between the Doppler parameters studied (CPUR, CPR and mUtA-PI) and umbilical arterial pH value could be observed.

Systematic meta-analyses already confirmed the association between low CPR and APO in low-risk pregnancies. However, corresponding to our results, an overall mild APO prediction rate was reported [9, 10]. The optimal cut-off value in defining CPR pathology is currently discussed (< 5th centile, < 10th centile, < 20th centile, multiple of the median, < 1.1) as well as the best time of CPR measurement [7–9, 24].

The idea of integrating the maternal side of the placenta into the feto-placental Doppler-based APO risk assessment built the foundation stone for first studies that examined the role of CPUR in high- and low-risk pregnancies [13–15]. Inclusion of mUtA-PI in the CPR to examine feto-placental unit might improve the APO prediction.

In a population of *n* = 347 patients, Macdonald et al. reported for the first time that the CPUR was the strongest indicator for a late mild placental dysfunction and predicted more cases of FGR (birth weight < 3rd centile) compared to the CPR and/or mUtA‐PI by itself [13]. A multicenter prospective study showed that in low-risk pregnancies (*n* = 804) at term, there was a six-fold increase in the rate of OD for IFC as well as a higher rate of APO in cases with a low CPUR, even though the predictive power of the CPUR was only moderate [14]. Morales-Rosello et al., however, could not observe an added predictive value of the CPUR with regard to APO when compared to the CPR between 34 + 0 and 41 + 0 weeks in a low-risk population (*n* = 891) [15].

According to the results of Dall’Asta et al. [14], the remaining placental reserve capacity at term, which might be represented by CPUR at best, could play a crucial role in the prediction of APO and OD for IFC in low-risk pregnancies. Uterine contractions during labor and the subsequent compression of the uterine arteries physiologically reduces the uteroplacental perfusion by up to 60% [25]. Hence, the antenatal placental function is crucial when it comes to the adequate fetal response to this physiological stress situation [12].

Looking at the progress made in the field of fetal monitoring, the examination methods (evaluation of fetal heart rate patterns, measuring amniotic fluid volume, estimating fetal weight) routinely used at full (39 + 0–40 + 6) or late (41 + 0–41 + 6) term [26] may be insufficient to detect subclinical PD [12]. Accordingly, the systematic investigation of established and novel Doppler indices in full and late term is important, although our results show a comparatively low predictive power of the CPUR regarding CAPO and OD for IFC. Recent data concerning the role of feto-maternal Doppler ultrasound in low-risk pregnancies and its association with APO, raise the question of whether abnormal Doppler indices, especially in the late-term situation (41 + 0–41 + 6), can or even should be cause for the recommendation of labor induction (not only an offer [16]) at 41 + 0 weeks of gestation (even in the case of normal amniotic fluid volume and CTG as well as an proven AGA-fetus) [12]. This is to be seen in particular against the background of the data of large randomized controlled trials on perinatal morbidity and mortality after induction of labor in the full and late-term period (vs. expectant management) [21, 22]. Hence, if feto-maternal Doppler ultrasound is routinely used as part of fetal monitoring in low-risk pregnancies at late term, an appropriate patient counseling and participative form of decision-making should be applied when deciding on the timing of labor induction [12].

There are several limitations of our study. As this is a retrospective observational study, the clinical consequence of a decreased CPUR like induction of labor and its influence on perinatal outcome, is not investigated. Thus, whether a change in CPUR is timely to achieve a reduction in the rate of emergency deliveries remains unresolved. Furthermore, the results need to be approved in prospective studies with increased numbers of cases. In particular, cerebral Doppler including MCA-PI measurement has been discussed as observer dependent [27]. Therefore, it would be desirable to have data of confirmed cerebro-placental-uterine Doppler pathology within 24 h to avoid false-positive results, especially when timing of delivery is based on this finding. We were not able to generate a threshold value for CPUR above which a CAPO was achieved, since too few cases were available for this purpose. Finally, there are (to our knowledge) no gestational age dependent percentile curves of the CPUR yet, which makes it difficult to give a clear statement about pathology and physiology.

In fact, the CAPO rate is comparatively high. In the differentiated consideration of the individual CAPO parameters, this is mainly due to the umbilical cord pH value and the NICU rate. We do not have an exact reason for this. However, we have chosen the inclusion criteria very strictly as we excluded women with conditions that may affect fetomaternal hemodynamics, especially such as those with maternal diabetes, pre-eclampsia or small-for-gestational age fetus.

A strength of the study is that, to our best knowledge, it is the first to examine CPUR in comparison to CPR in a clearly defined full-term collective (> 40 + 0 weeks). However, CPUR (similar to CPR) proved to be a poor predictor of adverse outcome. In order to integrate the CPUR into everyday clinical practice, published CPUR reference values and prospective evidence of a significant advantage in its use in the question of the ideal time of labor induction are required. The CPUR in > 40 + 0 weeks showed a mild predictive value for APO and OD due to IFC in low-risk pregnancies. However, the extent to which the CPUR (in comparison to the CPR) can be used to optimize delivery management based on its predictive value for OD for IFC warrants further investigations in prospective interventional studies.

## Supplementary Information

Below is the link to the electronic supplementary material.

Supplementary file1 (TIF 20777 KB) Graphical results of linear regression analysis for CPR, CPUR and mUtA-PI with arterial umbilical pH. CPR cerebroplacental ratio, CPUR cerebroplacental-uterine ratio, mUtA-PI mean uterine artery pulsatility index

Supplementary file2 (DOCX 14 KB)
