# Supplementary figures and images for: Role of the cerebro-placental-uterine ratio in predicting adverse perinatal outcome in low-risk pregnancies at term
Source: Arch Gynecol Obstet. 2022 Aug 30;308(3):849–55. doi: 10.1007/s00404-022-06733-8 (PMC10349005; doi:10.1007/s00404-022-06733-8)

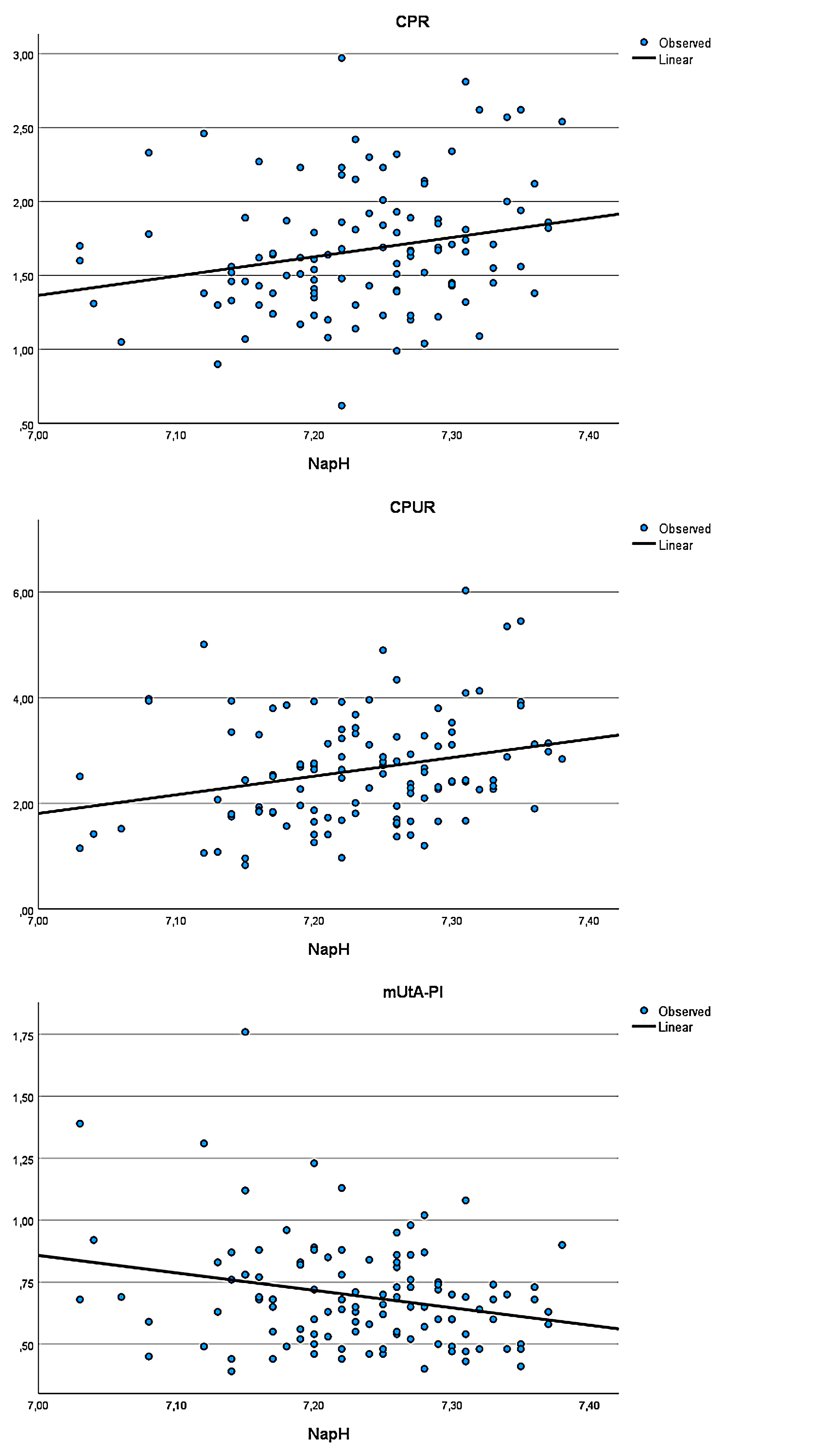

Supplement: Supplementary file 1 — Supplementary file1 (TIF 20777 KB) Graphical results of linear regression analysis for CPR, CPUR and mUtA-PI with arterial umbilical pH. CPR cerebroplacental ratio, CPUR cerebroplacental-uterine ratio, mUtA-PI mean uterine artery pulsatility index [file 404_2022_6733_MOESM1_ESM.tif]
