# Supplementary material for: Role of the cerebro-placental-uterine ratio in predicting adverse perinatal outcome in low-risk pregnancies at term
Source: Arch Gynecol Obstet. 2022 Aug 30;308(3):849–55. doi: 10.1007/s00404-022-06733-8 (PMC10349005; doi:10.1007/s00404-022-06733-8)
Supplement: Supplementary file 2 — Supplementary file2 (DOCX 14 KB) [file 404_2022_6733_MOESM2_ESM.docx]

| Parameter | Parameter Estimates | |
| --- | --- | --- |
|  | Sig. | b1 |
| CPR | 0.013 | 1.304 |
| CPUR | 0.005 | 3.523 |
| mUtA-PI | 0.008 | -0.704 |

Table S1: Numerical results of linear regression analysis for CPR, CPUR and mUtA-PI with arterial umbilical pH.
[CPR, cerebroplacental ratio; CPUR, cerebroplacental-uterine ratio; mUtA-PI, mean uterine artery pulsatility index; b1, unstandardized regression coefficient; Sig., Level of significance]
